# Axin1 Prevents *Salmonella* Invasiveness and Inflammatory Response in Intestinal Epithelial Cells

**DOI:** 10.1371/journal.pone.0034942

**Published:** 2012-04-11

**Authors:** Yong-guo Zhang, Shaoping Wu, Yinglin Xia, Di Chen, Elaine O. Petrof, Erika C. Claud, Wei Hsu, Jun Sun

**Affiliations:** 1 Department of Biochemistry, Rush University Medical Center, Chicago, Illinois, United States of America; 2 Department of Biostatistics and Computational Biology, University of Rochester, Rochester, New York, United States of America; 3 GI Diseases Research Unit and Division of Infectious Diseases, Department of Medicine, Queen's University, Kingston, Ontario, Canada; 4 Department of Pediatrics and Medicine, The University of Chicago Medical Center, Chicago, Illinois, United States of America; 5 Department of Biomedical Genetics, Center for Oral Biology, and James P Wilmot Cancer Center, University of Rochester, Rochester, New York, United States of America; 6 Gastroenterology and Hepatology Division, Department of Medicine, University of Rochester, Rochester, New York, United States of America; Charité, Campus Benjamin Franklin, Germany

## Abstract

**Background:**

Axin1 and its homolog Axin2 are scaffold proteins essential for regulating Wnt signaling. Axin-dependent regulation of Wnt is important for various developmental processes and human diseases. However, the involvement of Axin1 and Axin2 in host defense and inflammation remains to be determined.

**Methods/Principal Findings:**

Here, we report that Axin1, but not Axin2, plays an essential role in host-pathogen interaction mediated by the Wnt pathway. Pathogenic *Salmonella* colonization greatly reduces the level of Axin1 in intestinal epithelial cells. This reduction is regulated at the posttranslational level in early onset of the bacterial infection. Further analysis reveals that the DIX domain and Ser614 of Axin1 are necessary for the *Salmonella*-mediated modulation through ubiquitination and SUMOylation.

**Conclusion/Significance:**

Axin1 apparently has a preventive effect on bacterial invasiveness and inflammatory response during the early stages of infection. The results suggest a distinct biological function of Axin1 and Axin2 in infectious disease and intestinal inflammation while they are functionally equivalent in developmental settings.

## Introduction

Axin1 and 2 belong to the Axin family, a negative regulator of the Wnt signaling pathway and a key player in developmental processes and pathogenesis of human diseases [1], [2], [3], [4]. Axin forms a complex with GSK-3β an β-catenin and promotes GSK-3β-dependent phosphorylation of β-catenin [5], [6], [7]. In normal cells, β-catenin levels are kept low through interactions with GSK-3β, APC, and Axin [8], [9], [10], [11]. Dishevelled (Dsh) is the upstream regulator of the β-catenin pathway. At the C-terminal end, Axin can bind with Dsh and this interaction reduces β-catenin binding [8], [9], [10], [11]. Recently, more evidence demonstrates that Axin is involved in many other signaling pathways, including mTOR14, JNK MAPK15, parathyroid hormone16, and p53 signaling [10], [12], [13].

Bacterial infection is a stress to the host. Pathogens use a type three secretion system (TTSS) to inject bacterial pathogenic proteins, called effectors, into host cells [14]. Virulence bacterial effectors, such as *Salmonell*a AvrA, mimic the activity of a eukaryotic protein and debilitate their target cells [15]. Our publications and those of others demonstrate that β-catenin/Wnt [16], [17], [18], [19], [20], [21], [22], JNK/MAPK [15], [23], and p53 [24] are involved in bacterial infection and intestinal inflammation. Axin is a key player for the β-catenin/Wnt, JNK and p53 pathways. However, it is not known whether Axin directly regulates *Salmonella*-induced inflammation in the intestine.

In this current study, we investigated the molecular mechanism and physiological roles of Axin1-*Salmonella* interactions. We found that pathogenic *Salmonella* colonization decreased the Axin1 protein expression in intestinal epithelial cells at the post-transcriptional level. Bacterial protein AvrA expression stabilizes Axin1 protein and beta-catenin/Axin interactions. Axin1 exhibited a specific role in inhibiting *Salmonella* invasion and bacterial inflammation. It is affected by the level of Axin1, but not Axin2. The resulting data indicate an essential role of intestinal Axin1 in modulating host defense against pathogen-induced inflammation.

## Results

### Axin1 responds to *Salmonella* treatment

**Figure 1:**
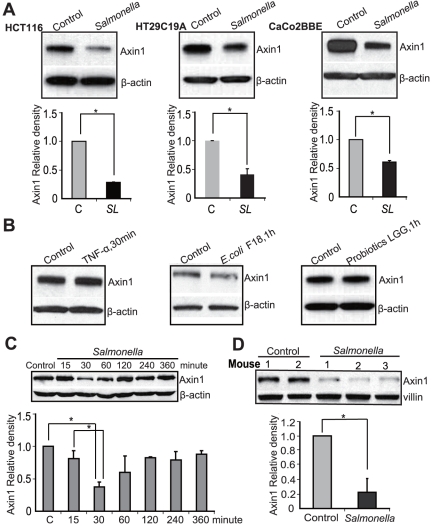
Pathogenic *Salmonella* decreases Axin 1 protein expression in host cells. (A) Axin1 protein expression in human epithelial cell lines. Cells were incubated with *S. typhimurium* wild-type (WT) for 30 minutes, washed, and incubated in fresh DMEM for 1 hour. Total cell lysates were analyzed for total Axin1 levels by immunoblot. *P<0.05. n = 3 separate experiments. (B) Inflammatory cytokine TNF-a, commensal bacterial *E. coli* F18, and probiotics L*GG* did not alter Axin1 protein expression in HCT116 cells. (C) *Salmonella* decreases Axin1 protein expression at the early stage of colonization in HCT116 cells. (D) Axin1 reduction by pathogenic *Salmonella* WT in intestinal epithelial cells *in vivo*. *Salmonella* decreases Axin1 protein expression in the mouse large intestine after bacterial infection *in vivo*. Data are expressed as mean ± SD.* P<0.05. n = 3 mice/group.

To determine whether Axin protein plays a role in epithelial-*Salmonella* interactions, we tested human intestinal epithelial HCT116 cells with wild-type (WT) *Salmonella* ATCC 14028s. We found that WT *Salmonella* significantly decreased the total amount of Axin1 in host cells after bacterial colonization for only 1 hour (Fig. 1A. HCT116). To look at the generality of our observation, we further investigated the response in the human colonic epithelial cell lines HT29C19A and CaCo2BBE. We had to use these cell lines because there is no non-cancer and non-transformed colon cell line available in the field. A similar change in Axin1 reduction by pathogenic WT *Salmonella* was found (Fig. 1A). To test if the response is specific to *Salmonella*, we also treated cells with inflammatory cytokine TNF-a, human commensal bacterial *E. coli* F18, and probiotic strain *Lactobacillus rhamnosus GG* (Fig. 1B). However, we did not see the similar alternation of Axin1. Furthermore, we found that the *Salmonella*-induced Axin1 reduction occurred in a time-dependent manner. WT *Salmonella* colonization of the cells for only 30 minutes was able to decrease Axin protein expression, and the effect could last for more than 60 minutes (Fig. 1C).

Using RT-PCR, we investigated Axin mRNA expression in intestinal epithelial cells. The transcriptional levels of Axin1 and 2 were not significantly changed by WT *Salmonella in vitro* (Fig. S1A and B). Overall, our data showed that pathogenic *Salmonella* reduces Axin1 protein.

### Reduction of Axin1 protein in the *Salmonella*-infected mouse intestine *in vivo*

Although cell lines provide useful information about the effects of *Salmonella* on Axin1 expression, these models lack the structural and biological relationships that exist *in vivo*. Therefore, we determined whether bacteria modulate Axin1 expression in streptomycin-pretreated C57BL6 mice colonized with bacterial strains. Streptomycin treatment is known to diminish the intestinal flora and to render mice susceptible to intestinal colonization by various microorganisms. In previous studies, we have established the streptomycin-pretreated *Salmonella*-colitis mice to understand the host-pathogen interactions [19], [20]. Post infection 8 hours is correlated with the early stage of *in vitro* bacterial colonization. Here, we found that Axin1 protein was significantly reduced by pathogenic *Salmonella* 8 hours postinfection, the early stage of infection *in vivo* (Fig. 1D). Additionally, the Axin1 mRNA level was not changed by *Salmonella* infection *in vivo* (Fig. S1C).

### Axin1 protein synthesis and degradation in epithelial cells colonized with *Salmonella*

**Figure 2:**
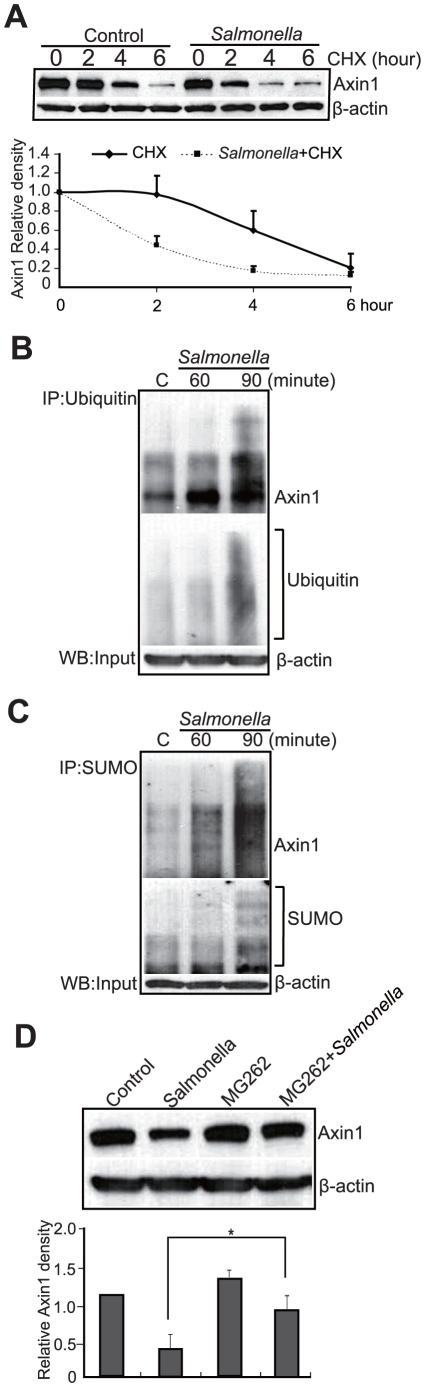
Axin1 reduction by *Salmonella* is through ubiquitination and SUMOylation. (A) Protein synthesis of Axin 1 is low in *Salmonella*-treated cells. (B) Ubiquitination of Axin1 increased in the *Salmonella*-colonized cells. (C) Sumoylation of Axin1 increased in the *Salmonella*-colonized cells. (D) Proteasome inhibitor MG262 stabilized Axin1 protein after *Salmonella* colonization. Cells treated with MG262 (20 µM) for 2 hours had increased levels of Axin1 protein. Data are expressed as mean ± SD. * P<0.05. n = 3 separate experiments.

To explore the molecular mechanism of *Salmonella*-induced Axin 1 reduction, we tested Axin1 protein synthesis using cycloheximide (CHX). CHX is an inhibitor of eukaryotic protein biosynthesis and is commonly used to determine protein half-life. Cells were treated with CHX; Axin1 protein synthesis was reduced after 4 hours of treatment without *Salmonella* (Fig. 2A). In contrast, Axin1 was dramatically reduced to 40% 2 hours after CHX and *Salmonella* treatment. A line chart further shows that the level of Axin1 protein synthesis was higher in untreated cells than in the *Salmonella*-treated cells. Hence, the rate of Axin1 degradation is faster in *Salmonella*-treated cells (Fig. 2A).

### Axin1 is regulated by *Salmonella* at the post-translational level

Because Axin protein destabilization occurred in the early stage of *Salmonella* invasion, we hypothesized that Axin1 was regulated at the post-transcriptional level upon *Salmonella* stimulation. It is known that Axin is phosphorylated and ubiquitinated and then is degraded by the proteasome [25]. Hence, we investigated whether *Salmonella* reduced Axin protein through increased ubiquitination. By Western blot assay, ubiquitinated protein appears as a smear of bands above the regular band of the target protein. We treated epithelial cells with *Salmonella* and tested Axin expression and ubiquitination using immunoprecipitation. Our data indicated that *Salmonella* treatment induced more ubiquitinated Axin1 compared to control cells. The ubiquitinated Axin1 (Ub-Axin) was enhanced after *Salmonella* colonization for 60 minutes (Fig. 2B).

Recent studies demonstrated that Axin is also regulated through SUMOylation [13], [25], [26]. We tested Axin1 SUMOylation in the *Salmonella*-treated epithelial cells. We found that the SUMOylated Axin1 was enhanced by treatment with WT *Salmonella* (Fig. 2C).

To determine whether Axin1 reduction occurs through increased proteasome degradation, cells were treated with the proteasome inhibitor MG262. In the presence of MG262, the level of Axin1 protein was comparable to that of the control cells (Fig. 2D). Taken together, these data showed that increased ubiquitination, SUMOylation, and proteasome degradation of Axin1 occurs during *Salmonella* infection.

### The amino acids specifically required for *Salmonella*-induced Axin1 degradation

**Figure 3:**
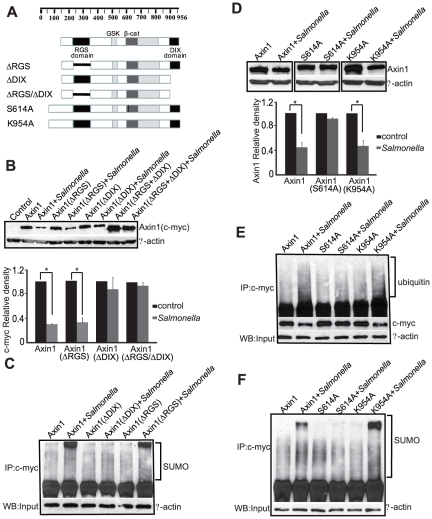
*Salmonella* target the Axin DIX domain and ubiquitination. (A) Diagrams of Axin1 constructs used in this study. (B) Protein levels of wild-type Axin1, Axin1 ΔRGS, Axin1 ΔDIX, and Axin1 ΔRGS ΔDIX mutations in cells with or without *Salmonella* colonization. (C) *Salmonella* target the ΔDIX mutations of Axin1 for SUMOlyation. (D) Axin1 mutants S614A and K954A had different responses to Salmonella colonization. Note that the Axin1 mutant S614A was not reduced by *Salmonella*. Data are expressed as mean ± SD. * P<0.05. n = 3 separate experiments. (E) Ubiquitination of AxinS614A did not increase in the *Salmonella*-colonized cells. (F) SUMOylation of Axin1 and its mutants S614A and K954 in the *Salmonella*-colonized cells.

To explore the molecular mechanism of Axin1 degradation in *Salmonella*-infected cells, we established an Axin overexpression system using HCT116 cells transiently transfected with a c-myc-tagged Axin1 protein (Fig. S2A). A sketch of the generated Axin1 mutations is shown in Fig. 3A. Axin1 contains a canonical regulator of G protein signaling (RGS) core domain and a DIX domain [8], [27]. We tested the Axin1 levels and its stabilization using these Axin1 mutants. We found that the levels of Axin1 ΔRGS domain mutant were significantly reduced after *Salmonella* infection (Fig. 3B). In contrast, the level of Axin1ΔDIX did not decrease after *Salmonella* infection. In addition, the Axin1 ΔRGS ΔDIX double mutation lost the target domain, and its protein level was not reduced by *Salmonella* infection. Moreover, IP data indicated that there was no change in Axin SUMOylation after *Salmonella* infection in cells transfected with Axin1 ΔDIX (Fig. 3C). In contrast, the SUMOylation of Axin ΔRGS was still enhanced by *Salmonella*.

We further tested whether *Salmonella* exploited Axin1 at key amino acid sites that regulate ubiquitination and SUMOylation. The Axin1S614A mutant lost its ability to be ubiquitinated and was not degraded by *Salmonella* (Fig. 3D). IP data indicated that *Salmonella* treatment did not change the ubiquitination of AvrA S614A, whereas K954A ubiquitination was enhanced by *Salmonella* (Fig. 3E). Moreover, *Salmonella* infection did not enhance Axin1S164A SUMOylation, whereas SUMOylation of Axin K954A was still high (Fig. 3F). We also tested the other Axin1 mutations (Fig. S3A); however, the protein levels of these Axin1 mutants did not change after *Salmonella* infection (Fig. S3B–D). Taken together, our data suggested that *Salmonella* targeted the ΔDIX domain of Axin1 and ser614 to reduce Axin1 protein level during host-bacterial interactions.

We also tested the function of Axin2 in the HCt116 cells. However, transfected Axin2 did not change after *Salmonella* treatment (Fig. S2C). In contrast, both endogenous Axin1 and exogenous c-myc tagged Axin1 decreased cells treated with WT *Salmonella* (Fig. S2B).

### Decreased β-catenin/Axin binding in *Salmonella* colonized cells

**Figure 4:**
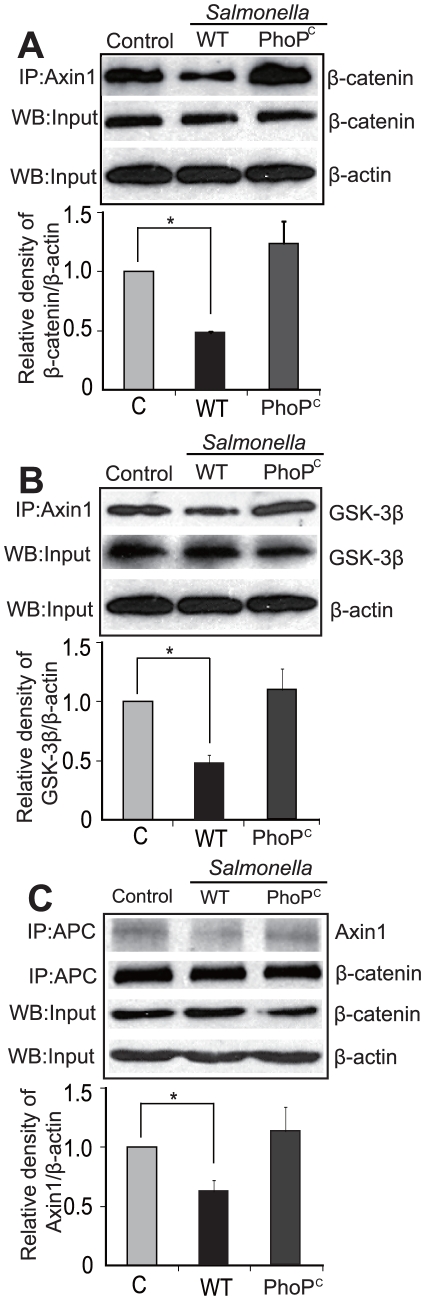
Axin/β-catenin interaction in cells infected with *Salmonella*. (A) Less Axin1 in a complex with β-catenin in cells infected by WT *Salmonella* compared to cells without bacterial treatment or with mutant Salmonella PhoPc. Data are expressed as mean ± SD. * P<0.05. n = 3 separate experiments. (B) Decreased Axin1/GSK-3β interaction in cells infected with WT *Salmonella* compared to cells without bacterial treatment or with mutant Salmonella PhoPc. Data are expressed as mean ± SD. * P<0.05. n = 3 separate experiments. (C) Reduced APC/Axin1 interaction in cells infected by WT *Salmonella* compared to cells without bacterial treatment or with PhoPc. Please note that the anti-APC antibody pulled down the same amount of β-catenin in cells with or without *Salmonella* colonization. It indicated that the APC/β-catenin interaction was not altered by *Salmonella*. Data are expressed as mean ± SD. * P<0.05. n = 3 separate experiments.

Axin can interact with many proteins in the cell [8]. It forms a complex with GSK-3β and β-catenin and promotes GSK-3β-dependent phosphorylation of β-catenin. Using immunoprecipitation and western blot, we found that WT *Salmonella* decreased the amount of Axin1 in a complex with β-catenin, whereas the binding between Axin1 and β-catenin was not altered by PhoP^C^ colonization (Fig. 4A). Densitometry analysis showed significant decreases in the binding of Axin1 and β-catenin after WT *Salmonella* colonization.

We also tested another Axin/β-catenin-associated protein, GSK-3β (Fig. 4B). The total amount of Axin bound to GSK-3βwas also reduced by WT *Salmonella*, whereas PhoP^c^ colonization stabilized the Axin/GSK-3β interaction within the cell. Furthermore, densitometry analysis showed a significant reduction of Axin/GSK-3β interaction induced by WT *Salmonella*.

WT *Salmonella* also reduced the amount of APC/Axin-binding proteins, whereas levels of the APC/β-catenin complex were not significantly changed after *Salmonella* infection (Fig. 4C). Taken together, these IP data indicated that pathogenic *Salmonella* colonization reduces Axin1 in the intestinal epithelial cells, thereby decreasing the dynamic interaction between Axin and its binding partners.

### Axin1 protein protects cells from *Salmonella* invasion

**Figure 5:**
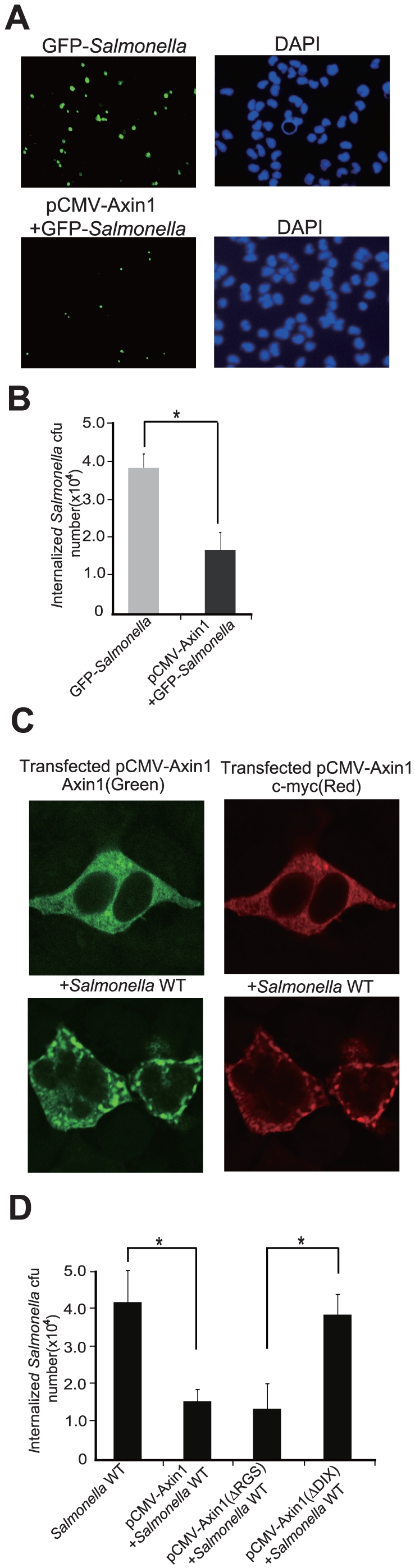
Physiologic function of Axin1 in host defense. (A) Axin1 expression in intestinal epithelial cells changed bacterial invasion. Cells overexpressing Axin1 were resistant to invasion of GFP-*Salmonella* (Green). (B) Number of bacteria associated with intestinal epithelial cells with different Axin1 levels. (C) Localization of Axin1 in the intestinal epithelial cells before and after bacterial infection. HCT 116 cells transfected with a c-myc-tagged Axin plasmid were colonized with *Salmonella*. Immunostaining showed that Axinl protein localized to the cell membrane and cytoplasm in HCT116 cells. Degraded Axin1 proteins were shown as green dots in cells infected with *Salmonella*. (D) Number of invasive bacteria in intestinal epithelial cells with different Axin1 mutations. Axin1 ΔDIX lost the effect to inhibit *Salmonella* invasion. Data are expressed as mean ± SD. * P<0.05. n = 3 separate experiments.

For the physiological relevance of Axin involved in the *Salmonella*-host interactions, a green-fluorescence-tagged *Salmonella* strain was used to detect bacterial invasion in intestinal epithelial cells. There was less green *Salmonella* in the cells with Axin1 overexpression compared to the normal cells (Fig. 5A). We further counted the numbers of *Salmonella* invading the HCT116 cells with normal or over-expressed Axin1 protein. We found that Axin1-over-expressing epithelial cells had less internalized *Salmonella* than control cells with regular Axin1 expression (Fig. 5B). We also examined the number of cell-associated bacteria, including bacteria adhered to and/or internalized into the epithelial monolayers. Axin1 expression did not change the number of associated *Salmonella* in the host cells (Fig. S4A).

We also investigated the distribution of Axin1. Immunostaining data showed that Axin1 protein was dispensed diffusely in the cytoplasm in HCT116 cells without bacterial treatment. Postpathogenic *Salmonella* infection, Axin1 was scattered as dots in the cytoplasm. Some Axin1 relocated to the cell membrane (Fig. 5C). Axin1 relocation to the cell membrane may help to block the bacterial invasion.

### The DIX domain is required for Axin1's function in inhibiting bacterial invasion

We investigated the effect of Axin1 mutations on bacterial invasion. As shown in Fig. 5D, cells with ΔRGS overexpression had less invaded *Salmonella*, whereas ΔDIX-expressing cells had a significantly higher amount of *Salmonella* invasion. Axin1 mutations did not change the number of bacteria associated with the epithelial cells (Fig. S4B). Taken together, these data further indicate that Axin1ΔDIX was targeted by *Salmonella*. Therefore, the mutated Axin1ΔDIX could not protect cells from bacterial invasion. However, Axin2 did not have any effects on *Salmonella* attachment and invasion (Fig. S5A & 5B).

### Axin1 protein is directly involved in *Salmonella*-induced inflammation

**Figure 6:**
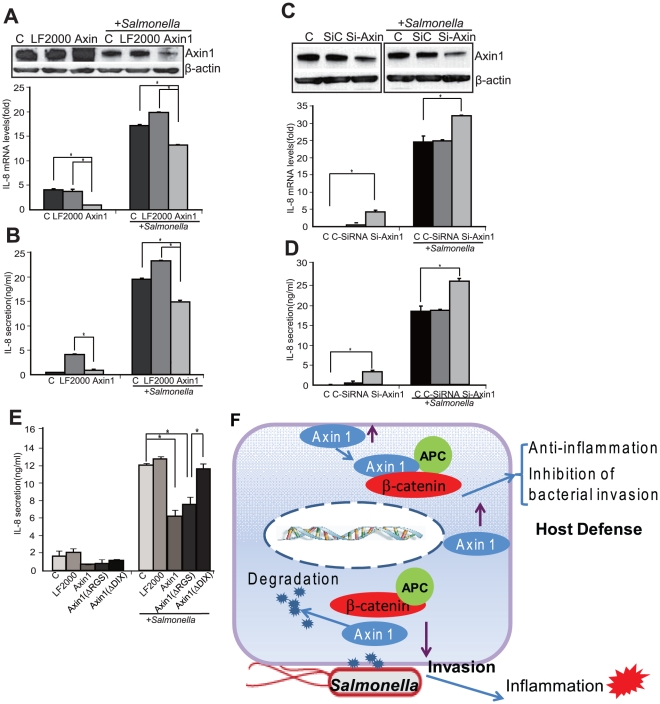
Axin1 expression in the intestinal epithelial cells changes inflammatory responses. Transient transfections were performed with Lipofectamine2000 (LF2000). (A) IL-8 mRNA in Axin1-overexpressing HCT116 cells after *Salmonella* colonization. (B) IL-8 protein secretion in cell culture media in Axin1-overexpressing HCT116 cells after *Salmonella* colonization. (C) IL-8 mRNA in cells with Axin1 blockage by siRNA of Axin1 overexpression and IL-8 protein. (D) *Salmonella*-induced IL-8 protein levels in cells with Axin1 blockage by using siRNA against Axin1. (E) *Salmonella*-induced IL-8 protein levels in cells with Axin1 mutations. Axin1 ΔDIX lost the effect to inhibit IL-8 secretion. Data are expressed as mean ± SD. * P<0.05. n = 3 separate experiments. (F) A diagram showing the roles of the Axin1 pathway in intestinal epithelial-*Salmonella* interactions. From bacterial side, *Salmonella* invasion induces degradation of Axin1 and dissociates Axin1/beta-catenin/APC complex in intestinal epithelial cells. From the host side, cells with high expression level of Axin1 are able to inhibit *Salmonella* invasion and suppress inflammation. Axin1 plays a novel role in host defense during bacterial-induced inflammation.

We reasoned that Axin1 expression could protect cells from *Salmonella*-induced inflammation. We tested the inflammatory response of IL-8 in cells overexpressing Axin1. Upon *Salmonella* colonization, IL-8 mRNA expression decreased in cells overexpressing Axin1 compared to normal HCT116 cells (Fig. 6A). We also found that the IL-8 protein secretion was significantly lower in cells overexpressing Axin1 (Fig. 6B). In addition, Axin2 overexpression did not have any effect on the expression of *Salmonella*-induced IL-8 at either the transcriptional (data not shown) or the translational level (Fig. S5C).

To further investigate the molecular mechanisms, we blocked Axin1 expression using siRNA. As shown in Fig. 6C–D, the knockdown of Axin1 significantly enhanced IL-8 mRNA expression and IL-8 protein secretion in the cell culture media compared with cells without treatment or with control siRNA. Moreover, the cells overexpressing Axin ΔRGS produced significantly less IL-8, whereas cells overexpressing ΔDIX had higher IL-8 secretion induced by *Salmonella* (Fig. 6E). Taken together, our data indicate that WT Axin1 plays a protective role in bacterial intestinal inflammation. The DIX domain is required for Axin1's functions.

## Discussion

In the current study, we showed that Axin1, but not Axin2, plays an important role in inhibiting bacterial invasion and inflammation in intestinal epithelial cells. The reduction of Axin1 occurred at an early stage of *Salmonella* infection (Fig. 6F). Axin1 protein was modified by *Salmonella*-colonization through ubiquitination and SUMOylation. Our findings further demonstrated that the protein expression level of Axin may determine the host's inflammatory responses to *Salmonella*. *Salmonella*-induced IL-8 was significantly lower in cells with Axin overexpression compared to those in the normal intestinal epithelial cells. In contrast, when Axin1 was knockdown, *Salmonella* induced significantly higher level of IL-8 expression. Axin1ΔDIX domain and ser164A is specifically required for *Salmonella*-induced degradation of Axin1. Although Axin 1 and 2 are functionally equivalent in developmental settings [28], [29], [30], a distinct biological function of Axin1 and 2 was identified in host defense. We reported that Axin2 was not involved in inhibiting bacterial invasion and inflammation, whereas Axin1 plays a protective role for host defense against pathogenic bacteria.

Our previous studies demonstrated that Wnt/β-catenin is involved in *Salmonella*-induced intestinal inflammation [19], [20], [21], [22], [31]. Classically, Axin1 is viewed as a negative regulator of the Wnt/β-catenin pathway in development and cancer. The degradation of Axin1 leads to activation of the Wnt/β-catenin pathway. The binding between β-catenin and Axin is modified by *Salmonella* colonization. This may further explain our previous observation that the Wnt/β-catenin pathway is involved in *Salmonella-induced* intestinal inflammation. In *in vitro* studies, we used HCT116 cells with full length APC and HT29 and Caco2BBE with truncated APC. We found that the length of APC may influence the abundance of Axin1 in the cells. However, it does not change the effect of *Salmonella*-induced downregulation of Axin1. Recent studies demonstrate that Wnt/β-catenin signaling is regulated through both Axin-dependent proteolysis and Axin-independent proteolysis [32]. Hence, some of the effects we observed, such as Axin1 overexpression inhibiting *Salmonella* invasion, could be β-catenin-independent.

In Axin1 over-expressing cells, there was less *Salmonella*-induced IL-8 secretion. This finding indicates that Axin1 expression can protect cells from inflammation. Recently, studies have implied the Wnt signaling in the activation of proinflammatory mediators in inflammatory disorders [33]. Patients with inflammatory bowel diseases (IBD) have significantly higher expression of Wnts compared to non-IBD patients [34]. There was reduced expression of the Wnt-signaling pathway transcription factor TCF-4 in ileal Crohn's disease [35]. However, some of these studies have focused on the responses of monocytes to the inflammatory cytokines in the immune system through Wnts [36]. The effects and molecular mechanisms of Axin, the key scaffold of Wnt signaling, in regulating intestinal inflammation remain unknown.

Axin1 has been shown to interact with many critical proteins, including β-catenin, GSK3 β, LRP5, and DVL1 [25], [37], [38], [39]. In addition, Axin1 expression determines p53 activation in cell development [12], [40]. It is still unknown how Axin1 may synergistically regulate multiple signaling pathways, thereby altering the host response to bacterial infection. A limitation of the current work is that we could not use whole Axin1 knockout mice for further *in vivo* studies, because its phenotype in mice is lethal. The Axin1 conditional mutant mice are available now [41], we will focus on the physiological role of intestinal Axin in *in vivo* in anti-inflammation and anti-infection for future studies.

In summary, the current study determines the protective role of Axin1 in the *Salmonella* invasion and bacterial-induced inflammation. Our results indicate that a previously undefined role of Axin1 in host-pathogen interactions and inflammation.

## Materials and Methods

### Ethics statement

All animal work was approved by the University of Rochester University Committee on Animal Resources (UCAR) committee (UCAR 2007-065). If a mouse showed that it had aspirated fluid or significant body weight loss (10% or more), and did not die immediately, the mouse was humanely euthanized.

### Bacterial strains and growth condition

Bacterial strains used in this study included *Salmonella typhimurium* wild-type strain ATCC14028 (WT-SL) and non-pathogenic *Salmonella* mutant strain PhoP^c^ [42]. Non-agitated microaerophilic bacterial cultures were prepared as previously described [22].

### Cell culture

Human colonic epithelial HCT116, CaCo2BBE, and HT29C19A cells were maintained in DMEM supplemented with 10% fetal bovine serum (FBS), penicillin-streptomycin and L-glutamine. The rat small intestinal IEC-18 cell line was grown in DMEM (high glucose, 4.5 g/L) containing 5% FBS (vol/vol), 0.1 U/ml insulin, 50 µg/ml streptomycin, and 50 U/ml penicillin.

### Streptomycin pre-treated mouse model

Animal experiments were performed using specific-pathogen-free female C57BL/6 mice (Taconic, Hudson, NY) that were 6–7 weeks old, as previously described [20], [43], [44]. The protocol was approved by the University of Rochester University Committee on Animal Resources (UCAR). Water and food were withdrawn 4 hours before oral gavage with 7.5 mg/mouse of streptomycin (100 µl of sterile solution or 100 µl of sterile water in control). Afterwards, animals were supplied with water and food ad libitum. Twenty hours after streptomycin treatment, water and food were withdrawn again for 4 hours before the mice were infected with 1×10^7^ CFU of *S. Typhimurium* (100 µl suspension in HBSS) or treated with sterile HBSS (control). Eight hours after infection, mice were sacrificed, and tissue samples from the intestinal tracts were removed for analysis.

### Mouse colonic epithelial cells

Mouse colonic epithelial cells were collected by scraping the mouse colon, including the proximal and distal regions. Cells were sonicated in lysis buffer (1% Triton X-100, 150 mM NaCl, 10 mM Tris pH 7.4, 1 mM EDTA, 1 mM EGTA pH 8.0, 0.2 mM sodium orthovanadate, protease inhibitor cocktail). The protein concentration was measured using BioRad Reagent (BioRad, Hercules, CA, USA).

### Transient transfections

Plasmids with c-myc-tagged, wild-type Axin1, Axin2, and Axin1 mutants were from Dr. Hsu's laboratory. Transient transfections were performed with Lipofectamine2000 (Invitrogen, San Diego, CA) in accordance with the manufacturer's instructions. At the indicated times after transfection, proteins were extracted with RIPA buffer (50 µM Tris-Hcl, pH 8.0 with 150 mM sodium chloride, 1.0% NP-40, 0.5% sodium deoxycholate, 0.1% SDS) for immunoblotting.

### Axin1 siRNA

HCT116 cells were grown in 12-well plates. The cells were transfected with on-Target plus smart pool human Axin 1 siRNA (Dharmacon Inc., Lafayette, MO) or scrambled siRNA control (Santa Cruz Biotechnology Inc., Santa Cruz, CA, USA) using Surefect reagent (SABiosciences, Frederick, MD). After transfection for 72 hours, cells were colonized by *Salmonella* for 30 min, washed, and incubated for 30 min in DMEM with Gentamicin (500 µg/ml), and then the levels of Axin and β-actin were assessed by western blot.

### Immunoblotting

Mouse epithelial cells were scraped and lysed in lysis buffer (1% Triton X-100, 150 mM NaCl, 10 mM Tris pH 7.4, 1 mM EDTA, 1 mM EGTA pH 8.0, 0.2 mM sodium orthovanadate, protease inhibitor cocktail), and then the protein concentration was measured. Cultured cells were rinsed twice in ice-cold HBSS, lysed in protein loading buffer (50 mM Tris, pH 6.8, 100 mM dithiothreitol, 2% SDS, 0.1% bromophenol blue, 10% glycerol), and then sonicated. Equal amounts of protein were separated by SDS-polyacrylamide gel electrophoresis, transferred to nitrocellulose, and immunoblotted with primary antibodies. The following antibodies were used: anti-Axin1 (Cell Signal, Beverly, MA, U.S.A), anti-APC, anti-Villin,anti-c-Myc (Santa Cruz Biotechnology Inc., Santa Cruz, CA, U.S.A.), anti-ubiquitin (Enzo life Science, 5120 Butler Pike Plymouth Meeting, PA, U.S.A.), anti-sumo1 (Boston Biochem, Cambridge, MA, USA), anti-GSK-3β, anti-β-catenin (1∶1000; BD, San Jose, CA, U.S.A.), or anti-β-actin (Sigma-Aldrich, Milwaukee, WI, U.S.A.) antibodies and were visualized by ECL. Membranes that were probed with more than one antibody were stripped before re-probing.

### Coimmunoprecipitation assay

Cells were rinsed twice in ice-cold HBSS and lysed in cold immunoprecipitation buffer (1% Triton X-100, 150 mM NaCl, 10 mM Tris·HCl, pH 7.4, 1 mM EDTA, 1 mM EGTA, pH 8.0, 0.2 mM sodium orthovanadate) containing protease inhibitor cocktail. Samples were precleared with protein A-agarose. Pre-cleared lysates were incubated with 2 µg of primary antibodies for 1 hour at 4°C. Protein A-agarose was added to the lysate and incubated for 30 min with agitation at 4°C before being washed with cold immunoprecipitation buffer. The pellet was resuspended in 0.1 M glycine, pH 2.5, and incubated with agitation for 10 min at 4°C and then centrifuged at 9,000 *g* for 2 minutes. The supernatant was removed and neutralized with 1 M Tris·HCl, pH 8.0. The samples were diluted with concentrated (5×) electrophoresis sample buffer (125 mM Tris, pH 6.8, 4% SDS, 10% glycerol, 0.006% bromophenol blue, 2% β-mercaptoethanol), boiled for 5 minutes, separated by SDS-polyacrylamide gel electrophoresis, and transferred onto a nitrocellulose membrane. Membrane blots were probed with secondary antibody and visualized by ECL.

### Immunofluorescence

Colonic tissues from the proximal and distal portion of the colon were freshly isolated and paraffin-embedded after fixation with 10% neutral buffered formalin. Immunofluorescence was performed on paraffin-embedded sections (1 µm) of mouse colons. After preparation of the slides as described previously [19], slides were incubated in 3% hydrogen peroxide for 20 minutes at room temperature to block endogenous peroxidase activity, followed by incubation for 20 minutes in 5% BSA with 0.1% saponin in PBS to reduce nonspecific background.

Cultured cells were rinsed three times in HBSS, fixed for 20 minutes in 100% cold ETOH, and permeabilized for 10 minutes with 0.2% Triton X-100, followed by three rinses with Hanks HBSS, and incubation for 30 minutes in 10% BSA in HBSS to reduce nonspecific background. The permeabilized cells or tissue samples were incubated with primary antibodies for 90 minutes at room temperature. Samples were then incubated with goat anti-rabbit Alexa Fluor 488 or goat anti-mouse Alexa Fluor 594 (Molecular Probes, CA; 1∶200) and DAPI (Molecular Probes 1∶10,000) for 1 hour at room temperature. Tissues or cells were mounted with SlowFade (SlowFade® AntiFade Kit, Molecular Probes), followed by a coverslip, and the edges were sealed to prevent drying. Specimens were examined with a Leica SP5 Laser Scanning confocal microscope.

### *S. typhimurium* invasion of human epithelial monolayers

Infection of HCT116 cells was performed by a previously described method [44], [45]. Bacterial solution was added, and bacterial invasion was assessed after 30 minutes. Cell-associated bacteria, representing bacteria adhered to and/or internalized into the monolayers, were released by incubation with 100 µl 1% Triton X-100 (Sigma). Internalized bacteria were those obtained from lysis of the epithelial cells with 1% Triton X-100 30 minutes after the addition of gentamicin (50 µg/ml). For both cell-associated and internalized bacteria, 0.9 ml LB broth was added, and each sample was vigorously mixed and quantitated by plating for CFU on MacConkey agar medium.

### *Salmonella*-induced human IL-8 secretion

HCT116 cells were cultured in DMEM, followed by *Salmonella*-containing HBSS for 30 minutes, washed 3 times in HBSS, and incubated at 37°C for 6 hours. Cell supernatants were removed and assayed for IL-8 by ELISA in 96-well plates.

### Real Time quantitative PCR

Total RNA was extracted from epithelial cell monolayers or mouse colonic epithelial cells using TRIzol reagent (Invitrogen, Carlsbad, CA). The RNA integrity was verified by gel electrophoresis. RNA reverse transcription was done using the iScript cDNA synthesis kit (Bio-Rad, Hercules, CA) according to the manufacturer's directions. The RT-cDNA reaction products were subjected to quantitative real-time PCR using the MyiQ single-color real-time PCR detection system (Bio-Rad) and iQ SYBR green supermix (Bio-Rad) according to the manufacturer's directions. All expression levels were normalized to β-actin levels of the same sample. Percent expression was calculated as the ratio of the normalized value of each sample to that of the corresponding untreated control cells. All real-time PCR reactions were performed in triplicate. All PCR primers were designed using Lasergene software (Table 1) (DNAStar, Madison, WI).

**Table 1:** Real-Time PCR Primers.

Gene name	Primers
Human Axin1-F	CCTGTGGTCTACCCGTGTCT
Human Axin1-R	GCTATGAGGAGTGGTCCAGG
Human Axin2-F	CTGGCTTTGGTGAACTGTTG
Human Axin2-R	AGTTGCTCACAGCCAAGACA
Mouse Axin1-F	ACGGTACAACGAAGCAGAGAGCT
Mouse Axin1-R	CGGATCTCCTTTGGCATTCGGTAA
Mouse Axin2-F	GAGTAGCGCCGTGTTAGTGACT
Mouse Axin2-R	CCAGGAAAGTCCGGAAGAGGTATG
Human IL8-F	TGCATAAAGACATACTCCAAACCT
Human IL-8R	AATTCTCAGCCCTCTTCAAAAA
Human β-actin-F	AGAGCAAGAGAGGCATCCTC
Human β-actin-R	CTCAAACATGATCTGGGTCA
Mouse β-actin-F	TGTTACCAACTGGGACGACA
Mouse β-actin-R	CTGGGTCATCTTTTCACGGT

### Statistical Analysis

Data are expressed as mean ± SD. Differences between two samples were analyzed by Student's t test. P-values of 0.05 or less were considered statistically significant. Differences among three or more groups were analyzed using ANOVA (SAS 9.2 version, SAS Institute Inc., Cary, NC).

## Supporting Information

Figure S1 **Pathogenic** ***Salmonella*** **decreases Axin 1 protein expression but not mRNA expression in the host cells.**
(PDF)

Figure S2 ***Salmonella*** **reduces Axin1 expression through ubiquitination and SUMOylation.**
(PDF)

Figure S3 **Protein levels of wild-type Axin1 and Axin1 mutants in cells with or without** ***Salmonella*** **colonization.**
(PDF)

Figure S4 **Axin1 expression in the intestinal epithelial cells did not change bacterial association.**
(PDF)

Figure S5 **Axin2 expression in the intestinal epithelial cells did not change bacterial infection and inflammatory responses.**
(PDF)
