# Supplementary material for: Axin1 Prevents Salmonella Invasiveness and Inflammatory Response in Intestinal Epithelial Cells
Source: PLoS One. 2012 Apr 11;7(4):e34942. doi: 10.1371/journal.pone.0034942 (PMC3324539; doi:10.1371/journal.pone.0034942)
Supplement: Figure S1 — Pathogenic Salmonella decreases Axin 1 protein expression but not mRNA expression in the host cells. (PDF) [file pone.0034942.s001.pdf]

Figure S1

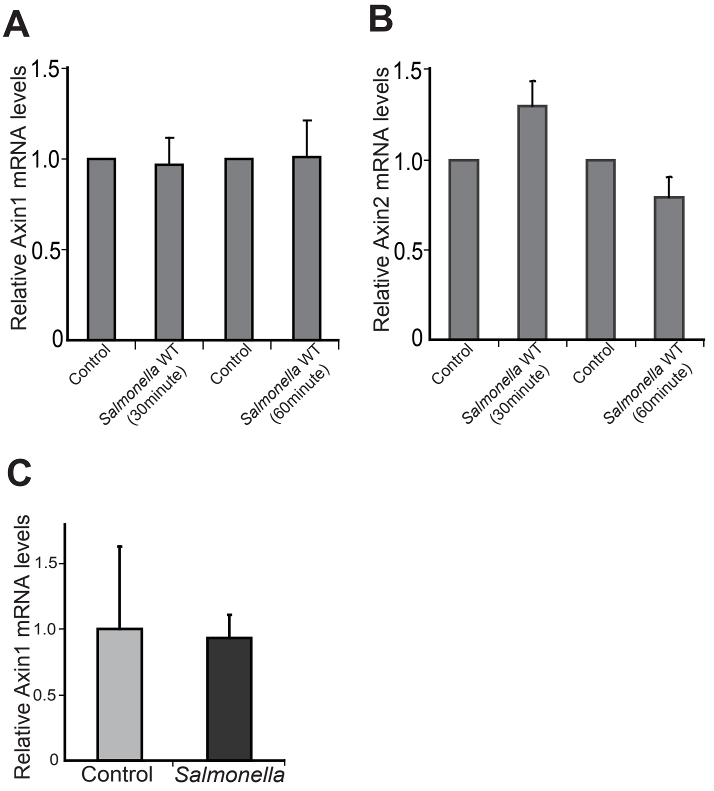

Figure S1. Pathogenic *Salmonella* decreases Axin 1 protein expression but not mRNA expression in the host cells. (A) Axin1 mRNA expression in intestinal epithelial HCT116 cells colonized with *Salmonella*. Cells were incubated with S. Typhimurium wild-type (WT) for 30 minutes, washed, and incubated in fresh DMEM for 1 hour. (B) *Salmonella* did not decrease Axin2 mRNA expression in HCT116 cells. (C) Axin1 mRNA is not altered by pathogenic WT *Salmonella* in intestinal epithelial cells in vivo. *Salmonella* did not decrease Axin1 mRNA expression in vivo. Data are expressed as mean ± SD. n=3 mice/group.
