# Supplementary material for: Axin1 Prevents Salmonella Invasiveness and Inflammatory Response in Intestinal Epithelial Cells
Source: PLoS One. 2012 Apr 11;7(4):e34942. doi: 10.1371/journal.pone.0034942 (PMC3324539; doi:10.1371/journal.pone.0034942)
Supplement: Figure S2 — Salmonella reduces Axin1 expression through ubiquitination and SUMOylation. (PDF) [file pone.0034942.s002.pdf]

Figure S2

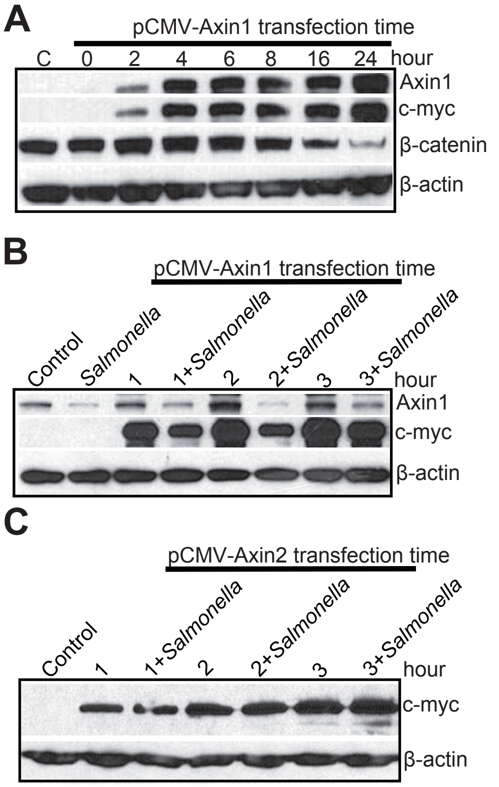

Figure S2. *Salmonella* reduces Axin1 expression through ubiquitination and SUMOylation. (A) Axin1 over-expression in intestinal epithelial cells. c-myc-tagged Axin1 in HCT116 cells after transfection for 24 hours. Note the decreased total β-catenin levels after Axin overexpression for 24 hours in the intestinal epithelial cells. (B) Axin1 reduction in the Axin1-overexpressing intestinal epithelial cells infected with wild-type (WT) *Salmonella* . C-myc-tagged Axin was expressed in HCT116 cells after transfection for only 3 hours. (C) Axin2 is not altered in the Axin2-overexpressing intestinal epithelial cells infected with wild-type *Salmonella* (WT).
