# Supplementary material for: Axin1 Prevents Salmonella Invasiveness and Inflammatory Response in Intestinal Epithelial Cells
Source: PLoS One. 2012 Apr 11;7(4):e34942. doi: 10.1371/journal.pone.0034942 (PMC3324539; doi:10.1371/journal.pone.0034942)
Supplement: Figure S3 — Protein levels of wild-type Axin1 and Axin1 mutants in cells with or without Salmonella colonization. (PDF) [file pone.0034942.s003.pdf]

Figure S3

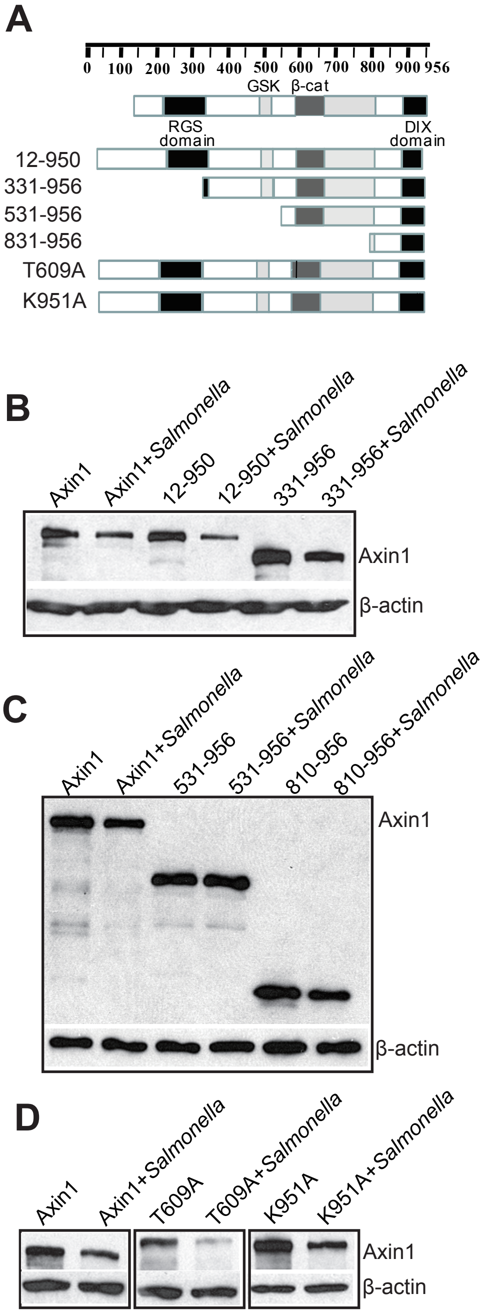

Figure S3. Protein levels of wild-type Axin1 and Axin1 mutants in cells with or without *Salmonella* colonization. (A) Diagrams of Axin1 mutant constructs. (B). Axin1 mutants (amino acid12-950 and 331-956) were reduced after *Salmonella* infection (WT). (C). Axin1 mutants (amino acid 531-956 and 810-956) were reduced after *Salmonella* infection. (D) Protein levels of wild-type Axin1, Axin1T609A, and K951A in cells with or without *Salmonella* colonization. Please note the reduction of wild-type Axin1 and Axin1 mutants after WT *Salmonella* colonization.
