# Supplementary material for: Axin1 Prevents Salmonella Invasiveness and Inflammatory Response in Intestinal Epithelial Cells
Source: PLoS One. 2012 Apr 11;7(4):e34942. doi: 10.1371/journal.pone.0034942 (PMC3324539; doi:10.1371/journal.pone.0034942)
Supplement: Figure S4 — Axin1 expression in the intestinal epithelial cells did not change bacterial association. (PDF) [file pone.0034942.s004.pdf]

Figure S4

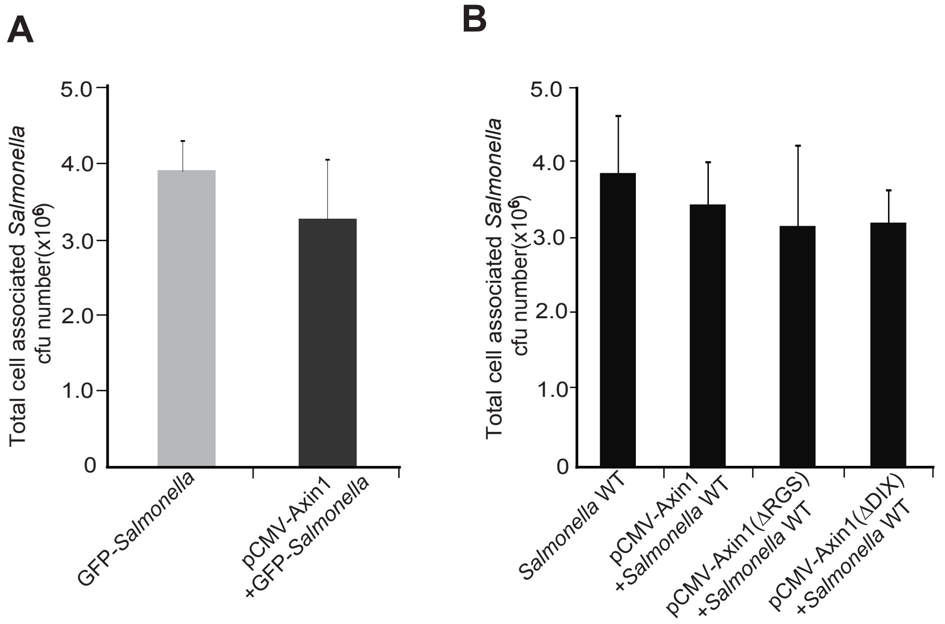

Figure S4. Axin1 expression in the intestinal epithelial cells did not change bacterial association. Cells overexpressing Axin1 had decreased invasion of *Salmonella* . (A) Number of *Salmonella* associated with intestinal epithelial cells with different Axin1 levels. (B) Number of bacteria associated with intestinal epithelial cells with different Axin1 mutations. Data are expressed as mean ± SD. n= 3 separate experiments.
