# Supplementary material for: Axin1 Prevents Salmonella Invasiveness and Inflammatory Response in Intestinal Epithelial Cells
Source: PLoS One. 2012 Apr 11;7(4):e34942. doi: 10.1371/journal.pone.0034942 (PMC3324539; doi:10.1371/journal.pone.0034942)
Supplement: Figure S5 — Axin2 expression in the intestinal epithelial cells did not change bacterial infection and inflammatory responses. (PDF) [file pone.0034942.s005.pdf]

Figure S5

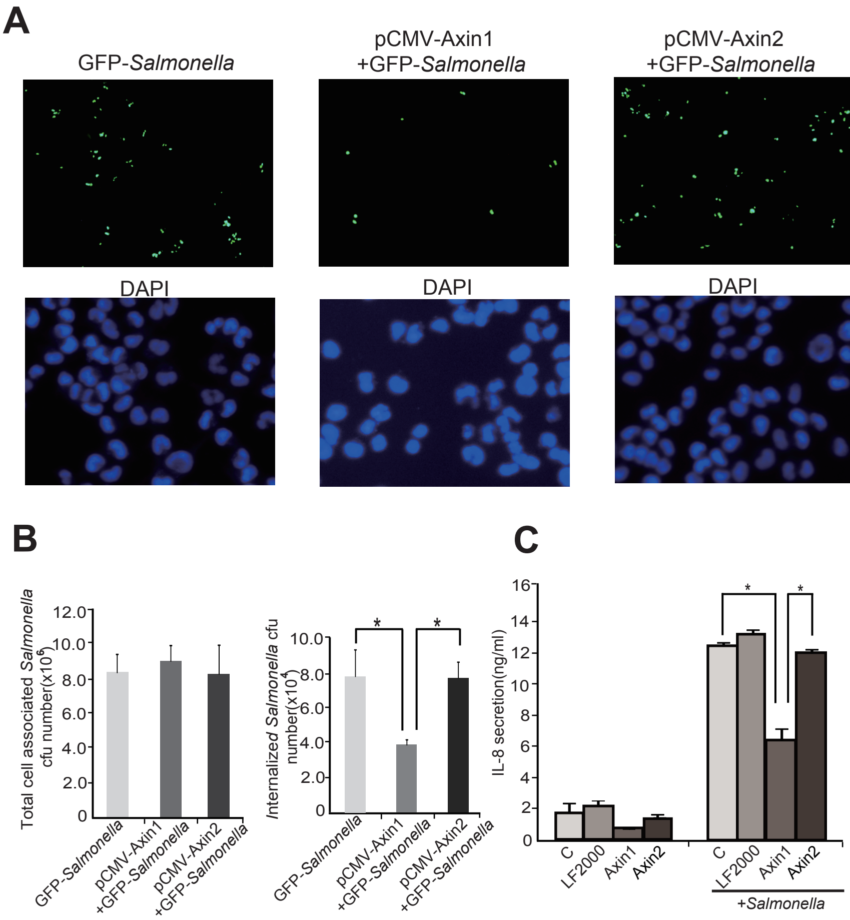

Figure S5. Axin2 expression in the intestinal epithelial cells did not change bacterial infection and inflammatory responses. (A) Cells overexpressing Axin2 had the same number of the GFP-*Salmonella* (Green) as those in the control cells with normal Axin2 level. However, Axin1 overexpression lowered bacterial invasion. (B) Number of bacteria associated with intestinal epithelial cells in Axin1 or 2-overexpressed HCT116 cells after *Salmonella* colonization. (C) IL-8 protein secretion in cell culture media in Axin1 or 2-overexpressing HCT116 cells after *Salmonella* colonization. Data are expressed as mean ± SD. * P< 0.05. n= 3 separate experiments.
